# Neonatal intrahepatic cholestasis caused by citrin deficiency with no hepatic steatosis: a case report

**DOI:** 10.1186/s12887-021-02717-w

**Published:** 2021-05-18

**Authors:** Ryosuke Miyamoto, Jun Sada, Koki Ota, Kenitiro Kaneko, Hironori Kusano, Yoshiteru Azuma, Akihisa Okumura

**Affiliations:** 1 Department of Pediatrics, Aichi Medical University School of Medicine, 1-1 Yazakokarimata, Nagakute, Aichi 480-1195 Japan; 2 Postgraduate Clinical Training Center, Aichi Medical University, Nagakute, Aichi Japan; 3 Department of Surgery, Aichi Medical University, Nagakute, Aichi Japan; 4 Department of Pathology, Kurume University School of Medicine, Kurume, Japan

**Keywords:** Neonatal intrahepatic cholestasis caused by citrin deficiency, Fatty liver, Genetic analysis

## Abstract

**Background:**

Neonatal intrahepatic cholestasis caused by citrin deficiency (NICCD) is a common form of neonatal jaundice. Histopathological examination of the liver in patients with NICCD typically shows fatty liver, steatohepatitis, and liver fibrosis. Jaundice and fatty liver often improve by 1 year of age. We herein describe a girl who was diagnosed with NICCD based on an *SLC25A13* mutation, although no fatty deposits were found on pathologic examination of the liver.

**Case presentation:**

The patient in this case was a 3-month-old girl. At 2 months of age, she presented with jaundice, discolored stools, and poor weight gain and was found to have hyperbilirubinemia. Cholangiography revealed that she did not have biliary atresia. A laparoscopic liver biopsy was performed, and liver histopathology showed no fatty deposits. Genetic analysis revealed a compound heterozygous mutation in *SLC25A13*, and she was diagnosed with NICCD. She was given medium-chain triglyceride milk and gained weight. She resumed consumption of normal milk and breast milk, and her stool color improved. She was discharged at 4 months of age with adequate weight gain and a lower total bilirubin concentration. She was in good condition after discharge and showed normal development at the time of outpatient follow-up.

**Conclusions:**

We experienced a case of NICCD in a patient without fatty liver. This case illustrates that the absence of hepatic steatosis in neonatal cholestasis does not rule out NICCD.

## Background

Neonatal intrahepatic cholestasis caused by citrin deficiency (NICCD) is a typical metabolic disorder that causes neonatal cholestasis. There are two clinical phenotypes of citrin deficiency: NICCD, which occurs from the neonatal to infantile period, and adult-onset type II citrullinemia (CTLN2), which occurs after puberty. In the period between these two forms, there is also a form called failure to thrive and dyslipidemia caused by citrin deficiency (FTTDCD), which is an adaptive and compensatory phase. The responsible gene is *SLC25A13*. The encoded protein, citrin, is an aspartate–glutamate carrier localized primarily in the mitochondrial inner membrane of the liver. This transport works as part of the malate aspartate shuttle. When aspartate is not transported into the cytoplasm due to citrin deficiency, the urea cycle is not levable to operate, and this causes citrulline levels to elevate. Loss of citrin function impairs urea and protein synthesis, aerobic glycolysis, glycogenesis, and energy metabolism, leading to a variety of symptoms. Histopathologic examination of the liver of children with NICCD generally shows fatty liver, steatohepatitis, hepatic fibrosis, and, rarely, cirrhosis. Cholestasis and fatty liver often improve by 1 year of age, but whether they later progress to CTLN2 remains to be determined. We herein describe a girl with NICCD diagnosed on the basis of variants in *SLC25A13*, although no fat deposition was seen upon pathological examination of the liver.

## Case presentation

The patient was a 3-month-old girl born at 40 weeks of gestational age with a birth weight of 2600 g. She was the first child of healthy non-consanguineous parents. She had no history of phototherapy for jaundice. Newborn Screening and regular 1-month checkup were unremarkable. At 2 months of age, the patient’s mother noticed jaundice and discolored stool and visited a local clinic. The infant’s weight gain had been insufficient during the last month. Her body weight remained around 4500 g despite the fact that she had been fully breastfed 8 to 10 times/day. She had icteric sclera and her skin was yellowish brown. Laboratory examinations showed a total bilirubin concentration of 9.74 mg/dl, direct bilirubin concentration of 5.64 mg/dl, and prothrombin time–international normalized ratio of 1.2. The patient was referred to our hospital after intravenous vitamin K infusion.

**Fig. 1:**
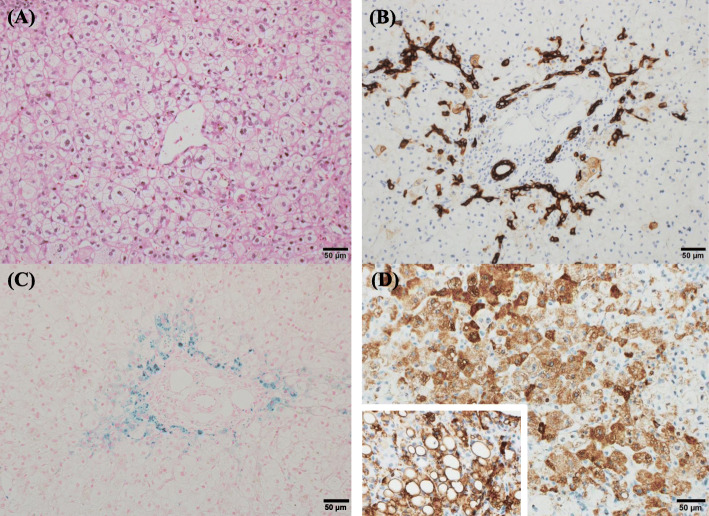
Pathological findings of liver tissue. **a** Hepatocyte swelling is notable. Bile plugs are also present. There is no fat deposition in the hepatocytes. **b** Cytokeratin 7 immunostaining shows a mild ductular reaction in the rim of the portal area and a clear interlobular bile duct. **c** Deposits of hemosiderin are found in the hepatocytes around the portal area. **d** Adipophilin immunostaining shows that the cytoplasm of the hepatocytes is positive, but perivacuolar staining is not highlighted (inset, positive control of fatty liver sample)

Although she was in good condition, yellowing of the sclera was evident and the skin was brownish in color. She excreted creamy grayish-white stools. Blood test data at the time of admission were AST 195 U/L, ALT 43 U/L, GGT 95 U/L, albumin 3.7 g/dl, AFP 35898.7 ng/ml, T-chol 196 mg/dl, and triglycerides 233 mg/dl. At the last follow-up at 13 months of age, the data were AST 69 U/L, ALT 29 U/L, GGT 83 U/L, albumin 4.5 g/dl, total cholesterol 208 mg/dl, and triglycerides 233 mg/dl. And no evidence of Epstein-Barr virus, Cytomegalovirus, Hepatitis A, B, and C virus was found in this case. Serum amino acid analysis showed a normal citrulline level of 39.9 nmol/ml and an elevated threonine concentration of 385.0 nmol/ml. The threonine/serine ratio was within the normal range. Abdominal ultrasonography confirmed the presence of a gallbladder and the absence of the triangular cord sign in the hilar region, and 24-h biliary scintigraphy showed poor excretion. Biliary atresia was ruled out by cholangiography. We performed laparoscopic liver biopsy. Macroscopically, the liver was generally yellow due to cholestasis. Pathological examination demonstrated cholestasis and hydropic changes, no obvious inflammatory cell infiltration within the lobule, no evidence of fat deposition (Fig. 1a), and a mild ductular reaction in the margins of the portal area as confirmed by cytokeratin 7 immunohistochemistry (Fig. 1b). The bile ducts were adequate in number, and the interlobular bile duct count/portal vein count ratio was about 0.7. Berlin blue staining revealed iron (hemosiderin) deposition in the periportal hepatocytes (Fig. 1c). Immunostaining for adipophilin, a fatty droplet-binding protein, showed positive staining in the cytoplasm of the hepatocytes; however, the perivacuolar areas were not clearly stained, indicating no evidence of steatosis (Fig. 1d).

Genetic analysis revealed compound heterozygous variants in *SLC25A13*: NM_014251.3, c.674C > A(p.Ser225*), and c.1801G > T(p.Glu601*). The infant’s father and mother were found to be carriers of each mutation. She was diagnosed with NICCD and treated with ursodeoxycholic acid. Her bilirubin concentration gradually declined and her jaundice improved. Her stool color returned to normal. She was fed medium-chain triglyceride milk and her body weight increased. At 5 months of age, she resumed consumption of normal milk and breast milk, and her stool color improved. Her body weight exceeded 5000 g, and her total bilirubin concentration decreased to around 2 mg/dl. She was discharged from the hospital at 4 months of age. She was in good condition after discharge and showed normal psychomotor development at the last follow-up at 13 months of age.

## Discussion and conclusions

NICCD is an autosomal recessive disease that causes intrahepatic cholestasis in newborns. There are two major categories of NICCD: one in which a positive newborn screening (high levels of galactose, methionine, and phenylalanine) reveals liver damage, and the other in which newborn screening is normal and the patient is diagnosed with prolonged jaundice and poor weight gain after 1 month of age [1]. The present case falls into the latter category.

NICCD is known to be often associated with fatty liver on pathological examination. Remarkably, however, fatty liver was not present in our patient. Kimura et al. [2] reported the pathological findings of 30 patients with NICCD; 29 patients showed fatty liver, and the fatty deposits were especially severe in 20 patients. Only one patient had no hepatic steatosis. However, detailed information on the genetic mutations, symptoms, and outcomes was not available [2]. Inui et al. [3] described an 8-year-old girl with an *SLC25A13* mutation who showed no fatty changes of the liver by light microscopy. In this patient, very small fatty droplets were observed by electron microscopy [3]. Although we did not evaluate our patient by electron microscopy, no fatty deposits were observed upon immunostaining for adipophilin. We consider that fatty changes of the liver were absent in our patient.

Komatsu et al. [4] reported that the expression of peroxisome proliferator-activated receptor alpha (PPARα), a master regulator of hepatic lipid metabolism, was significantly reduced in the liver tissue of patients with CTLN2. The expression of hepatic PPARα was inversely correlated with the severity of steatosis and the citrin concentration, indicating that down-regulation of PPARα was associated with fatty liver in patients with CTLN2. The expression of hepatic PPARα should be further investigated in patients with NICCD to clarify the reason for the different severity of fatty liver.

At present, the genotype–phenotype correlation of *SLC25A13* is unclear. We found a combination of two nonsense variants (S225X and E601X) in our patient. We presume that homozygous or compound heterozygous situations of nonsense variants can cause NICCD. For instance, homozygous or compound heterozygous situations of two mutations (c.851del4 and IVS11L1G > A) are caused both early- and late-onset citrin deficiency [5]. Although we did not find the same combination of variants in previous reports as in our patient, each variant caused phenotypes of both NICCD and CTLN2 [5–8]. We experienced a case of NICCD without fatty liver. NICCD should always be included in the differential diagnosis of cholestasis in newborns. This case suggests that NICCD should not be ruled out even if hepatic steatosis is not seen in newborns with cholestasis.

## Data Availability

The data that support the findings of this case report are available from the corresponding author upon reasonable request.

## Abbreviations

NICCD: Neonatal intrahepatic cholestasis caused by citrin deficiency
CTLN2: Adult-onset type II citrullinemia
PPARα: Peroxisome proliferator-activated receptor alpha

## References

[1] Ohura T, Kobayashi K, Tazawa Y, Abukawa D, Sakamoto O, Tsuchiya S, Saheki T (2007). Clinical pictures of 75 patients with neonatal intrahepatic cholestasis caused by citrin deficiency (NICCD). J Inherit Metab Dis.

[2] Kimura A, Kage M, Nagata I, Mushiake S, Ohura T, Tazawa Y, Maisawa S, Tomomasa T, Abukawa D, Okano Y (2010). Histological findings in the livers of patients with neonatal intrahepatic cholestasis caused by citrin deficiency. Hepatol Res.

[3] Inui A, Hashimoto T, Sogo T, Komatsu H, Saheki T, Fujisawa T (2016). Chronic hepatitis without hepatic steatosis caused by citrin deficiency in a child. Hepatol Res.

[4] Komatsu M, Kimura T, Yazaki M, Tanaka N, Yang Y, Nakajima T, Horiuchi A, Fang ZZ, Joshita S, Matsumoto A (2015). Steatogenesis in adult-onset type II citrullinemia is associated with down-regulation of PPARalpha. Biochim Biophys Acta.

[5] Yamaguchi N, Kobayashi K, Yasuda T, Nishi I, Iijima M, Nakagawa M, Osame M, Kondo I, Saheki T (2002). Screening of SLC25A13 mutations in early and late onset patients with citrin deficiency and in the Japanese population: identification of two novel mutations and establishment of multiple DNA diagnosis methods for nine mutations. Hum Mutat.

[6] Kobayashi K, Sinasac DS, Iijima M, Boright AP, Begum L, Lee JR, Yasuda T, Ikeda S, Hirano R, Terazono H (1999). The gene mutated in adult-onset type II citrullinaemia encodes a putative mitochondrial carrier protein. Nat Genet.

[7] Hayasaka K, Numakura C, Toyota K, Kimura T (2012). Treatment with lactose (galactose)-restricted and medium-chain triglyceride-supplemented formula for neonatal intrahepatic cholestasis caused by citrin deficiency. JIMD Rep.

[8] Togawa T, Sugiura T, Ito K, Endo T, Aoyama K, Ohashi K, Negishi Y, Kudo T, Ito R, Kikuchi A (2016). Molecular Genetic Dissection and Neonatal/Infantile Intrahepatic Cholestasis Using Targeted Next-Generation Sequencing. J Pediatr.

